# Additive effect of LRP8/APOER2 R952Q variant to APOE ε2/ε3/ε4 genotype in modulating apolipoprotein E concentration and the risk of myocardial infarction: a case-control study

**DOI:** 10.1186/1471-2350-10-41

**Published:** 2009-05-13

**Authors:** Nicola Martinelli, Oliviero Olivieri, Gong-Qing Shen, Elisabetta Trabetti, Francesca Pizzolo, Fabiana Busti, Simonetta Friso, Antonella Bassi, Lin Li, Ying Hu, Pier Franco Pignatti, Roberto Corrocher, Qing Kenneth Wang, Domenico Girelli

**Affiliations:** 1 Department of Clinical and Experimental Medicine, University of Verona, Verona, Italy; 2 Department of Molecular Cardiology, Centre for Cardiovascular Genetics, Department of Cardiovascular Medicine, Cleveland Clinic, and Department of Molecular Medicine, Cleveland Clinic Lerner College of Medicine of Case Western Reserve University, Cleveland, Ohio, USA; 3 Section of Biology and Genetics, Department of Mother and Child and Biology – Genetics, University of Verona, Verona, Italy; 4 Institute of Clinical Chemistry, University of Verona, Verona, Italy

## Abstract

**Background:**

The R952Q variant in the low density lipoprotein receptor-related protein 8 (LRP8)/apolipoprotein E receptor 2 (ApoER2) gene has been recently associated with familial and premature myocardial infarction (MI) by means of genome-wide linkage scan/association studies. We were interested in the possible interaction of the R952Q variant with another established cardiovascular genetic risk factor belonging to the same pathway, namely apolipoprotein E (APOE) ε2/ε3/ε4 genotype, in modulating apolipoprotein E (ApoE) plasma levels and risk of MI.

**Methods:**

In the Italian cohort used to confirm the association of the R952Q variant with MI, we assessed lipid profile, apolipoprotein concentrations, and APOE ε2/ε3/ε4 genotype. Complete data were available for a total of 681 subjects in a case-control setting (287 controls and 394 patients with MI).

**Results:**

Plasma ApoE levels decreased progressively across R952Q genotypes (mean levels ± SD = RR: 0.045 ± 0.020, RQ: 0.044 ± 0.014, QQ: 0.040 ± 0.008 g/l; *P *for trend = 0.047). Combination with APOE genotypes revealed an additive effect on ApoE levels, with the highest level observed in RR/non-carriers of the E4 allele (0.046 ± 0.021 g/l), and the lowest level in QQ/E4 carriers (0.035 ± 0.009 g/l; *P *for trend = 0.010). QQ/E4 was also the combined genotype with the most significant association with MI (OR 3.88 with 95%CI 1.08–13.9 as compared with RR/non-carriers E4).

**Conclusion:**

Our data suggest that LRP8 R952Q variant may have an additive effect to APOE ε2/ε3/ε4 genotype in determining ApoE concentrations and risk of MI in an Italian population.

## Background

A genome-wide linkage scan in combination with case control association analysis has become an effective strategy for studying the genetics of complex diseases because it is an unbiased approach without a priori hypothesis to identify susceptibility genes. The low density lipoprotein receptor-related protein 8 (LRP8) gene, located on chromosome 1p34-36 [1], is an example of successful use of this type of approach in discovering genes for coronary artery disease (CAD) and myocardial infarction (MI). The rs5174 polymorphism of LRP8 gene, coding for the nonconservative substitution R952Q, has been associated with familial CAD and MI in different independent populations [2], but not in sporadic MI populations [2,3]. The LRP8 protein is a lipoprotein receptor belonging to the low-density lipoprotein receptor (LDLR) family, and expressed in several different cells and tissues, e.g. brain, testes, heart, endothelial cells, smooth muscle cells, and platelets [4].

LRP8 is also known as apolipoprotein E receptor 2 (ApoER2) [5]. Apolipoprotein E (ApoE) is the main ligand for receptor-mediated clearance of VLDLs and chylomicron remnants, thus contributing to modulation of plasma levels of lipoproteins, cholesterol and triglycerides (TG) [6]. Among several proposed susceptibility genes for CAD/MI, the apolipoprotein E (*APOE *located on chromosome 19q13.2) gene was one of the few survivors, as recently confirmed by a meta-analyses involving thousands of subjects [7]. Two common APOE polymorphisms (Cys_112_Arg and Arg_158_Cys) define the APOE ε2/ε3/ε4 variants (APOE2 or ε2 = Cys_112_/Cys_158_; APOE3 or ε3: Cys_112_/Arg_158_; APOE4 or ε4 = Arg_112_/Arg_158_), and have been demonstrated to modulate cardiovascular risk [8]. This may occur either directly through modulation of plasma levels of ApoE, which may be involved in atherogenesis *per se*, and/or through modulation of plasma lipid profile because of the different ApoE receptor-binding affinities of the three isoforms (APOE4>APOE3>APOE2) [6]. Consequently, APOE4 is generally associated with lower levels of ApoE and higher levels of ApoB and cholesterol, as well as with an increased cardiovascular risk [7,9,10].

In light of the common pathway shared by APOE and LRP8/APOER2 genes, we were interested in investigating the possible combined effect of LRP8 R952Q and APOE ε2/ε3/ε4 variants in influencing ApoE levels and/or cardiovascular risk. This hypothesis was tested by a case-control association study design within the cohort of the Verona Heart Study (VHS).

## Methods

### Study Population

The VHS is an ongoing study aimed to identify new risk factors for CAD and MI in a population of subjects with documented angiographic data on coronary vessels. Details about the enrolment criteria have been described previously [11]. In the present study, we examined the genotyping data on LRP8 and APOE from the cohort of subjects previously used to replicate the association of LRP8 R952Q with cardiovascular risk [2]. Within this cohort (n = 724) we analysed APOE genotypes and the plasma concentration of ApoE, as well as the plasma lipid profile. A total of 681 subjects, enrolled between May 1996 and March 2000, for whom complete analyses were available, were included in this study. Of these, 394 had angiographically documented severe coronary atherosclerosis (being candidates to coronary artery bypass grafting) and a history of previous MI (MI group), documented by combining data from clinical history with a thorough review of medical records showing diagnostic electrocardiogram and enzyme changes, and/or the typical sequelae of MI on ventricular angiography. Two hundred eighty-seven subjects with completely normal coronary arteries, being evaluated by coronary angiography for reasons other than CAD (mainly valvular heart disease) were considered as controls (CAD-free group). Controls were also required to have neither history nor clinical or instrumental evidence of atherosclerosis in vascular districts beyond the coronary bed. At the time of blood sampling, a complete clinical history was collected, including the assessment of cardiovascular risk factors such as obesity, smoking, hypertension and diabetes, as well as medication and therapy.

The study was approved by the local Ethical Committee. Informed consent was obtained from all the subjects after a full explanation of the study.

### Biochemical analysis

Samples of venous blood were drawn from each subject after an overnight fast. Serum lipids and the other common biochemical parameters were determined by routine methods. LDL levels were estimated by means of the Friedewald equation. ApoA1, ApoB and ApoE were measured by commercially available nephelometric immunoassays; antisera, calibrators and the BNII nephelometer were from Dade Behring. ApoC-III was measured by a fully automated turbidimetric immunoassay as previously described [12]. The reagents were obtained from Wako Pure Chemical Industries, and the procedure recommended by the manufacturer was implemented on a RxL Dimension Analyzer (Dade International Inc.).

### Genotyping of polymorphisms

Genomic DNA was extracted from whole blood samples by the phenol-chloroform procedure. For LRP8 R952Q polymorphism, high-throughput SNP genotyping was performed using the 5' nuclease allelic discrimination assay (TaqMan Assay) on an ABI PRISM 7900HT Sequence Detection System as previously described [2].

APOE ε2/ε3/ε4 variants were genotyped according to a previously described multilocus assay [13], except for the use of a simplified PCR protocol to improve APOE genotyping, which was validated by RFLP and sequencing. Briefly, each sample was amplified by two 33-cycle multiplex polymerase chain reactions (PCR, 32 ng of genomic DNA each) and the PCR products were then hybridized to an array of immobilised, sequence-specific oligonucleotide probes. The colorimetric detection was based upon streptavidin-horseradish peroxidase.

### Statistical analysis

All statistical analyses were performed with SPSS 14.0 statistical package (SPSS Inc., Chicago, IL). Distributions of continuous variables in groups were expressed as means ± standard deviation. Logarithmic transformation was performed on all skewed variables, including TG, ApoC-III and ApoE. Therefore, the statistical differences concerning these parameters were also computed on the corresponding log-transformed values, although, for the sake of clearness, non-transformed data were reported in the Results. Quantitative data were assessed using the Student's t-test or by ANOVA with polynomial contrasts for linear trend when indicated. Associations between qualitative variables were analyzed with the χ^2^-test, and linear trend analysis when indicated. The frequencies of the LRP8 and APOE genotypes were compared with the values predicted on the basis of the Hardy-Weinberg equilibrium by χ^2^-test. To avoid an excessive subfractioning of the study population in the analysis of combined genotypes (LRP8 R952Q stratified for APOE ε2/ε3/ε4 polymorphism), APOE genotype stratification was simplified by considering two main groups, i.e. carriers E4 and non-carriers E4. Moreover, a linear regression model estimating standardized beta-coefficients was performed to assess the role of LRP8 and APOE genotype as independent determinants of serum ApoE levels. A value of *P *< 0.05 was considered significant.

To assess the extent to which genetic polymorphisms were associated with MI, odds ratios with 95% CIs were estimated by univariate logistic regression analysis. Adjustment for classical cardiovascular risk factors (i.e. sex, age, hypertension, smoke, diabetes, cholesterol and triglyceride) was performed by adding those covariates in a multiple logistic-regression model. Analyses of potential interactions between LRP8 R952Q and APOE ε2/ε3/ε4 variants in determining ApoE levels and MI risk were performed by means of linear and logistic regression, respectively.

## Results

The general characteristics of the study population consisting of controls and MI patients are summarized in Table 1. As expected, the classical cardiovascular risk factors were more represented among MI patients. As previously reported, the R952Q variant was associated with MI, with homozygous carriers of Q allele showing a near two-fold increased risk (QQ *versus *RR homozygotes: OR = 1.92 with 95% CIs 1.19–3.12 by univariate analysis, and OR = 1.83 with 95% CIs 1.003–3.34 by multiple logistic regression after adjustment for classical cardiovascular risk factors, i.e. sex, age, hypertension, smoke, diabetes, cholesterol and triglycerides). On the other hand, APOE4 variant was slightly – but not significantly – more represented among MI patients (OR 1.39 with 95%CI 0.92–2.1 for carriers E4 *versus *non-carriers E4).

**Table 1:** General characteristics of the study population with myocardial infarction (MI) and controls

**Characteristics**	**Controls^a^** **(n = 287)**	**MI^a^** **(n = 394)**	***P***
**Age (years)**	58.1 ± 12.6	59.9 ± 10.0	0.048 *
**Male sex (%)**	69.0	84.0	<0.001 ^#^
**BMI (kg/m^2^)**	25.3 ± 3.4	26.6 ± 3.3	<0.001 *
**Hypertension (%)**	34.3	62.4	<0.001 ^#^
**Smoking (%)**	44.9	70.8	<0.001 ^#^
**Diabetes (%)**	6.2	16.2	<0.001 ^#^
**Total cholesterol (mmol/L)**	5.54 ± 1.08	5.80 ± 1.17	0.005 *
**LDL-cholesterol (mmol/L)**	3.58 ± 0.95	3.94 ± 0.99	<0.001 *
**HDL-cholesterol (mmol/L)**	1.42 ± 0.40	1.19 ± 0.29	<0.001 *
**Triglycerides (mmol/L)**	1.51 ± 0.67	1.99 ± 1.09	<0.001 *
**Apo A-I (g/l)**	1.42 ± 0.27	1.28 ± 0.22	<0.001 *
**Apo B (g/l)**	1.08 ± 0.26	1.23 ± 0.31	<0.001 *
**Apo C-III (mg/dl)**	10.8 ± 3.2	12.2 ± 4.6	<0.001 *
**ApoE (g/l)**	0.043 ± 0.014	0.046 ± 0.023	0.107
**LRP8 R952Q polymorphism (%)**			
**RR**	43.9	38.1	
**RQ**	45.3	43.9	0.027 ^#^
**QQ**	10.8	18.0	
**APOE ε2/ε3/ε4 polymorphism (%)**			
**carrier E2**	8.7	8.6	
**E3/E3**	76.7	72.1	0.280 ^#^
**carrier E4**	14.6	19.3	

^a ^Only 681 subjects with complete analyses of LRP8 R962Q genotypes, APOE genotypes, and the ApoE plasma concentration were included.

*: by t-test

^#^: by χ^2^-test

**Figure 1:**
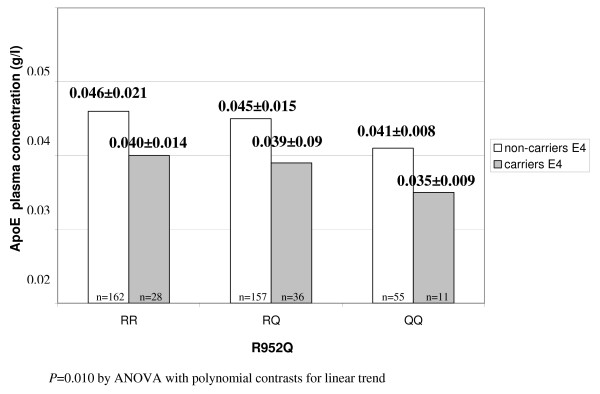
**Additive effect of LRP8 R952Q and APOE ε2/ε3/ε4 polymorphisms in determining plasma ApoE levels in non-diabetic subjects without lipid-lowering therapy (n = 449)**.

To assess the relation between genotypes and lipid profile without potential confounding factors, we excluded from the analysis subjects with diabetes (known to be associated with high levels of triglyceride-rich lipoproteins) or those taking lipid-lowering drugs. In this subgroup (n = 449), the ApoE plasma concentration decreased progressively across R952Q genotypes from "wild-type" RR homozygotes to QQ homozygotes (RR: 0.045 ± 0.020, RQ: 0.044 ± 0.014, QQ: 0.040 ± 0.008 g/l; *P *for trend = 0.047; Table 2). As expected, APOE ε2/ε3/ε4 genotype was confirmed as an important determinant of lipid profile, strongly influencing plasma ApoE, ApoB, total and LDL-cholesterol plasma concentrations (Table 3). In a linear regression analysis, both LRP8 and APOE genotype were independent predictors of ApoE levels also after adjustment for disease status, (standardized beta-coefficient = -0.095, *P *= 0.049, and -0.123, *P *= 0.011, respectively). When analyzed in combination, we found an additive effect of LRP8 and APOE variants in determining the plasma ApoE concentration, with a progressive decrease from the highest level in RR/non-carriers E4 to the lowest level in QQ/carriers E4 (*P *for trend = 0.010; Figure 1). More precisely, the interaction between LRP8 and APOE variants was not significant by regression analysis (*P *= 0.566), suggesting a reciprocally independent influence on ApoE levels of the 2 polymorphisms that act additively in determining the plasma ApoE concentration (i.e. the presence of one genotype does not influence the effect of the other on ApoE levels). On the other hand, no additive effect or interaction between the two gene variants was found with respect to other parameters of lipid profile (data not shown).

**Table 2:** Lipid profile according to LRP8 R952Q in non-diabetic subjects without lipid-lowering therapy (n = 449)

**LRP8 R952Q variant**	**RR** **(n = 190)**	**RQ** **(n = 193)**	**QQ** **(n = 66)**	P *
**Total cholesterol (mmol/l)**	5.63 ± 1.10	5.90 ± 1.15	5.68 ± 0.90	0.774
**LDL-cholesterol (mmol/l)**	3.75 ± 0.98	3.92 ± 1.02	3.84 ± 0.80	0.546
**HDL-cholesterol (mmol/l)**	1.30 ± 0.37	1.36 ± 0.39	1.27 ± 0.37	0.643
**Triglyceride (mmol/l)**	1.63 ± 0.83	1.74 ± 0.86	1.70 ± 0.92	0.530
**Apo A-I (g/l)**	1.34 ± 0.25	1.36 ± 0.26	1.31 ± 0.25	0.426
**Apo B (g/l)**	1.13 ± 0.29	1.18 ± 0.27	1.17 ± 0.32	0.129
**Apo C-III (mg/dl)**	10.9 ± 3.2	11.5 ± 3.8	11.1 ± 3.7	0.825
**ApoE (g/l)**	0.045 ± 0.020	0.044 ± 0.014	0.040 ± 0.008	0.047

*: by ANOVA with polynomial contrasts for linear trend

**Table 3:** Lipid profile according to APOE ε2/ε3/ε4 polymorphism in non-diabetic subjects without lipid-lowering therapy (n = 449)

**APOE ε2/ε3/ε4 variant**	**Carrier E2** **(n = 41)**	**E3/E3** **(n = 333)**	**Carrier E4** **(n = 75)**	P *
**Total cholesterol (mmol/l)**	5.40 ± 1.18	5.73 ± 1.08	6.01 ± 1.10	0.005
**LDL-cholesterol (mmol/l)**	3.34 ± 0.85	3.85 ± 0.97	4.03 ± 0.96	0.002
**HDL-cholesterol (mmol/l)**	1.34 ± 0.37	1.31 ± 0.37	1.37 ± 0.45	0.457
**Triglyceride (mmol/l)**	1.97 ± 1.36	1.65 ± 0.76	1.69 ± 0.91	0.562
**Apo A-I (g/l)**	1.36 ± 0.25	1.34 ± 0.25	1.36 ± 0.25	0.976
**Apo B (g/l)**	0.98 ± 0.31	1.16 ± 0.28	1.24 ± 0.28	<0.001
**Apo C-III (mg/dl)**	12.7 ± 5.7	11.0 ± 3.3	11.1 ± 2.9	0.307
**ApoE (g/l)**	0.067 ± 0.037	0.042 ± 0.010	0.039 ± 0.011	<0.001

*: by ANOVA with polynomial contrasts for linear trend

**Figure 2:**
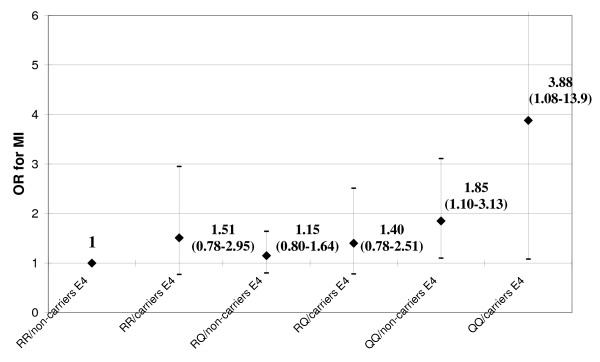
**Risk of MI in relation to LRP8 R952Q and APOE ε2/ε3/ε4 polymorphisms in the whole study population (n = 681)**. RR/non-carriers E4 are considered as reference group for calculating OR.

In the whole study population, there was also an additive effect of LRP8 and APOE variants on the risk of MI (*P *= 0.007 by χ^2^-test for linear trend), i.e. with the highest risk in QQ/carriers E4 (OR 3.88 with 95%CI 1.08–13.9 as compared with RR/non-carriers E4 – Figure 2). This association with MI remained significant even after adjustment for age and sex (OR 4.19 with 95%CI 1.13–15.6), whereas the statistical significance was marginally lost in a regression adjusted for all the traditional atherosclerosis risk factors (OR 3.84 with 95%CI 0.80–18.6). Similarly with the previous analysis, the interaction was not significant by regression analysis (P = 0.739), suggesting that the two polymorphisms act additively also in determining the risk of MI.

## Discussion

The LRP8 variant R952Q variant has been associated with an increased risk of CAD. Previously we reported that the R952Q variant was associated with increased platelet aggregation, which may be through increased phosphorylation of p38 mitogen-activated protein kinase (MAPK), providing a possible functional explanation for increased vascular risk [2]. Moreover, recent studies have confirmed a role of LRP8 in platelet activation through modulation of signal transduction of some ligands, like protein C, activated protein C and dimeric form of β 2-glycoprotein I [14,15]. On the other hand, the results of the present study suggest that, in addition to the previously demonstrated platelet hyperaggregation [2], the LRP8/APOER2 R952Q variant may influence the cardiovascular risk also through modulation of the ApoE pathway, particularly in combination with another genetic variant in the same pathway, APOE ε2/ε3/ε4. These two genetic variants may have an additive effect on both ApoE concentrations and the risk of MI.

With respect to the modulation of circulating ApoE levels, some lines of evidence support the biological plausibility of the interaction between the two genetic variants. LRP8/APOER2 protein is in fact not only an important modulator of platelet signalling [16], but also a lipoprotein receptor, with five functional domains closely related to those of LDL- and VLDL-receptors. Notwithstanding these similarities, ApoER2 appears to bind with high affinity only to ApoE-rich β-VLDL, while affinity for LDL and other VLDL is much lower [5]. The binding leads to internalization of ApoE containing lipid vesicles [17], and thus it is conceivable that functional genetic variants of LRP8/APOER2 may contribute to modulation of ApoE levels. The R952Q variant has been already shown to be a "gain-of-function" variant since it was associated with increased phosphorylation of p38 MAPK in platelets stimulated by oxidized LDL [2]. Indeed, we found a limited and specific influence of the R952Q variant on the ApoE plasma concentration, with lower levels in QQ homozygotes. It is tempting to speculate that the glutamine to arginine substitution at position 952 may also increase internalization of ApoE by modifying the receptor-affinity for ApoE, but further biochemical studies are needed to verify this hypothesis. No significant association was found between the R952Q variant and other lipid parameters, consistent with the elective affinity of LRP8/APOER2 for ApoE.

On the other side of the receptor/ligand interaction, it is of interest to note that the well-known APOE ε2/ε3/ε4 variant is known to modulate the affinity of the ApoE protein for lipoprotein receptors. Remarkably, APOE4, the isoform with the highest binding affinity for lipoprotein receptors, has been associated with the lowest ApoE plasma concentration [6]. Thus, the lowest ApoE levels we found in subjects carrying the compound genotype APOER2-952QQ/APOE4 is in accordance with the possibility of the additive combination of a high-affinity ligand with a receptor with high internalization activity.

Interestingly, in our study population the two genetic variants of the ApoE pathway showed an additive effect also in terms of association with the risk of MI, which increased progressively with the highest OR in carriers with combined APOER2-952QQ/APOE4. The APOE4 variant is indeed one of the few alleles of candidate genes which has been consistently confirmed to be a risk factor of CAD/MI, particularly through comprehensive meta-analyses involving thousands of subjects [7,9,10]. In the present study, E4 carriers were marginally more represented in MI patients without reaching statistical significance probably because of the relatively small sample size. The increased cardiovascular risk of APOE4 variant is generally considered to be a consequence of high LDL-cholesterol level [6]. However, some findings challenge this interpretation as the sole explanation of APOE genotype-associated atherogenic risk. In a prospective study, the increased risk of cardiovascular events in E4 carriers has been found to be unrelated to their cholesterol level [18]. Besides its involvement in the clearance of VLDLs and chylomicron remnants, ApoE has been implicated in other functions with potential anti-atherogenic properties, such as regulation of vascular smooth muscle cell proliferation, inhibition of platelet aggregation, anti-oxidant properties, anti-inflammatory activity, and regulation of immune response [6]. Based on these findings, it has been proposed that low ApoE levels may represent a cardiovascular risk factor *per se *(e.g., independent of plasma lipids) [19]. Though this hypothesis is not universally accepted [20], it may explain the particularly increased risk of MI found in carriers with combined APOER2-952QQ/APOE4, i.e. in subjects genetically predisposed to the lowest ApoE levels. On the other hand, in the current study there was no significant difference of ApoE concentrations between MI patients and controls. This apparent paradox may be, at least in part, explained by the fact that ApoE is localized on VLDLs and chylomicron remnants. Thus, ApoE plasma levels may also be a surrogate for plasma levels of the atherogenic triglyceride-rich and remnants lipoprotein particles [19,20].

## Conclusion

Our data suggest that LRP8 R952Q variant may have an additive effect to APOE ε2/ε3/ε4 genotype in determining plasma ApoE concentrations and the risk of MI in an Italian population. This study suffers from common limitations of genetic association studies of complex traits, particularly with a relatively small sample size. For example, the loss of significance in the last regression model about the risk of MI seems likely to be due to the small sample size of the subgroup analysis. Noteworthy, there was not a substantial decrease of the OR value, but rather a widened CI range [21]. Nevertheless, our finding represents a hypothesis-generating example of a gene-gene additive effect in the same metabolic pathway that warrants further future replications to assess the combined effects of APOER2/APOE variants on modulation of the risk of MI.

## Abbreviations

APOE: apolipoprotein E gene; ApoE: apolipoprotein E; ApoER2: apolipoprotein E receptor 2; CAD: coronary artery disease; LDLR: low-density lipoprotein receptor; LRP8: lipoprotein receptor-related protein 8; MAPK: mitogen-activated protein kinase; MI: myocardial infarction

## Competing interests

The authors declare that they have no competing interests.

## Authors' contributions

NM and DG: study design, statistical analysis, and writing the manuscript. GQS, ET, AB, LL, YH, and PFP: molecular biology and laboratory analysis, and review of the manuscript. FP, FB and SF: patients' selection and recruitment, data analysis, and review of the manuscript. OO, RC, and QKW: coordination of study design, and drafting the manuscript.

## Pre-publication history

The pre-publication history for this paper can be accessed here:


